# ﻿*Sillago persica* sp. nov., a new sillaginid (Perciformes, Sillaginidae) from the Persian Gulf

**DOI:** 10.3897/zookeys.1255.162247

**Published:** 2025-10-09

**Authors:** Hashem Khandan Barani, Mohammad Sadegh Alavi-Yeganeh, Mehdi Ghanbarifardi

**Affiliations:** 1 Department of Aquatic sciences, Hamoun International Wetland Institute, Research Institute of Zabol, Zabol, Iran Tarbiat Modares University Nur Iran; 2 Department of Marine Biology, Faculty of Natural Resources and Marine Silences, Tarbiat Modares University, Nur, Iran Hamoun International Wetland Institute, Research Institute of Zabol Zabol Iran; 3 Department of Biology, Faculty of Science, University of Sistan and Baluchestan, Zahedan, Iran University of Sistan and Baluchestan Zahedan Iran

**Keywords:** New species, sillaginids, *Sillago persica*, taxonomy

## Abstract

A new species of sillaginid fish, *Sillago persica***sp. nov**., is described from 30 specimens collected along the southern coast of the Persian Gulf (Busher), Iran. Morphological comparisons with 15 congeneric species, including members of the *S. sihama* complex, show that *S. persica* closely resembles *S. sihama* in meristic counts and coloration but is clearly distinguished by a unique swim bladder morphology. The posterior sub-extensions of the anterolateral swim bladder extensions in *S. persica* display a complex structure comprising five broad extensions connected to the swim bladder body, a feature absent in *S. sihama* and other sillaginids. Additional diagnostic characters include distinct meristic, morphometric, and coloration traits. Molecular analyses of mitochondrial cytochrome c oxidase subunit I (COI) sequences corroborate this species’ distinctiveness, with phylogenetic reconstruction placing *S. persica* haplotypes and a previously recognized G5 clade haplotype of the *S. sihama* complex in a strongly supported monophyletic clade sister to *S. indica* and *S. suezensis.* Intraspecific genetic divergence within haplotypes of silaginid species and clades of *S. sihma* ranged from 0.00 to 0.01 (K2P). The *S. persica* haplotypes matched the G5 clade of *S. sihama* (0.01, K2P distance) whereas it exhibited 0.18–0.24 K2P divergence from haplotypes of other sillaginid species and clades of the *S. sihama* complex. A detailed morphological description is provided along with diagnostic illustrations.

## ﻿Introduction

Members of the family Sillaginidae Richardson, 1846 are widely distributed in inshore waters characterized by sandy substrates and estuarine environments throughout the Indo-West Pacific region (McKay 1992). Commonly known as sand whitings or sand borers, these small to medium-sized demersal marine fishes are of considerable commercial and recreational importance throughout their range (McKay 1992; Gray and Kennelly 2003). Currently, five genera, *Sillago* Cuvier, 1816, *Sillaginopodys* Fowler, 1933, *Sillaginops* Kaga, 2013, *Sillaginopsis* Gill, 1861, and *Sillaginodes* Gill, 1861, comprising 39 valid species are recognized within the family (Saha et al. 2024). Recent taxonomic efforts have expanded knowledge of this group through the description of new species and revision of their geographic distributions (Cheng et al. 2021; Saha et al. 2022; Yu et al. 2022). Notably, four recently described species, *Sillago nigrofasciata*Xiao et al., 2021, *Sillago parasihama*Yu et al., 2022, *Sillago muktijoddhai*Saha et al., 2022, and *Sillago mengjialensis*Saha et al., 2022, were previously misidentified as *S. sihama* (Xiao et al. 2021; Saha et al. 2022; Yu et al. 2022). *Sillago sihama* is the most extensively sampled species in the family and lives in diverse habitats across the Indo-West Pacific; however, it is known to be a cryptic species complex (Cheng et al. 2021).

Species identification within the family Sillaginidae is often challenging due to the highly similar external morphological characters. This has led to the misidentification of biologically distinct species and the synonymization of valid taxa under widespread species (Krück et al. 2013). Among diagnostic characters, the morphology of the swim bladder is especially useful for distinguishing sillaginid species. Based on swim bladder structure, McKay (1985) proposed three subgenera within the genus *Sillago: Sillaginopodys* Fowler, 1933, characterized by a reduced swim bladder lacking a duct-like process; *Sillago* Cuvier, 1817 with two tapering posterior extensions and a duct-like process; and *Parasillago* McKay, 1985, which exhibits a swim bladder with a single posterior extension and a duct-like process. While this subgeneric classification aids in the taxonomy and phylogenetic analysis of *Sillago*, the similarity of swim bladder morphology among some closely related species (e.g. *S. sihama* and *S. shaoi*) complicates species-level identification, necessitating the use of additional morphological or molecular evidence.

DNA barcoding has been increasingly applied for rapid and accurate species identification and discovery of cryptic species within various fish groups, including sillaginids (Cheng et al. 2021; Xiao et al. 2021; Mulvaney et al. 2023). Notably, Cheng et al. (2021) identified eight cryptic lineages within the *S. sihama* complex across the Indo-West Pacific region by integrating phenotypic traits with molecular data, including mtDNA gene fragments (12S, 16S, COI, Cytb) and a nuclear gene fragment (Rag2). In addition, the phylogenetic placement of the *Sillaginopodys chondropus* haplotype within *Sillago* haplotypes in this study highlights the importance of a comprehensive taxonomic approach that combines detailed morphological examination with molecular analyses to resolve species boundaries and taxonomic issues in sillaginids. In the present study, we describe a new *Sillago* species from the Persian Gulf coast, based on an integrative approach that includes morphometric and meristic characters, swim bladder morphology, vertebral counts, and mitochondrial cytochrome c oxidase subunit I (COI) gene sequences.

## ﻿Materials and methods

### ﻿Sampling

**Figure 1.**
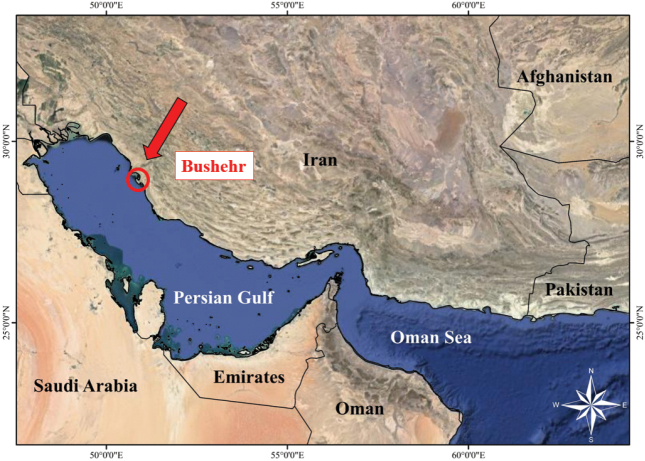
Sampling site of *Sillago persica* in the Persian Gulf.

Specimens of the newly described species were obtained directly from local fishermen and through fishery surveys conducted along the coast of Bushehr city in the northern Persian Gulf, Iran (Fig. 1). The holotype and paratype specimens have been deposited in the Aquatic Animals collection at the Department of Marine Biology, Tarbiat Modares University, Nur, Iran (TAC1245F and TAC1246F).

### ﻿Morphological analysis

The genus and species classification follows McKay (1985, 1992). Terminology for the appendages of the swim bladder adheres to the descriptions provided by Shao et al. (1986), Kaga and Ho (2012), and Cheng et al. (2021). Gill rakers and swim bladders were examined in the dissected paratypes. The identification of various vertebrae was based on the descriptions by McKay (1992). In addition to the swim bladder and vertebrae, 10 meristic and 17 morphometric characters were analyzed, along with descriptive traits such as body and fin coloration. All measurements were taken with dial calipers to the nearest 0.1 mm. Standard length (SL) and head length (HL) are abbreviated accordingly.

### ﻿Genetic analysis

Mitochondrial DNA (mtDNA) cytochrome oxidase subunit I (COI) fragments were amplified using the primers FishF1: 5′-TCAACCAACCACAAAGACATTGGCAC-3′ and FishR1: 5′-TAGACTTCTGGG TGGCCAAAGAATCA-3′ (Ward et al. 2005). A polymerase chain reaction (PCR) was conducted according to the protocols established by Gao et al. (2011).

The cleaned DNA underwent sequencing in a cycle sequencing reaction. Sequences were then assembled, aligned, and edited using BIOEDIT v. 7.0.4 and MAFFT v. 7. The nine COI sequences obtained for this research have been deposited in GenBank with accession numbers PV523932–PV523934 (*S. sihama*), PV524004, PV524005 (*S. arabica*), PV524006 (*S. attenuata*), and PV524007–PV524009 (*S. persica*). An additional 63 sequences from the Sillaginidae were downloaded from GenBank (Fig. 4) including haplotypes representing eight clades of the *S. sihama* complex (Cheng et al. 2021). This was done to create a comprehensive dataset for evaluating the phylogenetic position of the new sequences in this study and for reconstructing the phylogenetic relationships among representatives of the Sillaginidae. According to Cheng et al. (2021), two haplotypes from *Terapon jarbua* were selected as the outgroup.

The initial phylogenetic analysis was conducted using the maximum-likelihood (ML) method through IQ-TREE v. 1.6.12 (Nguyen et al. 2015). To assess branch support, we employed the UFboot2 bootstrap test with 10,000 replicates (Hoang et al. 2018). The evolutionary model TPM2+F+I+G4 was identified as the best-fit model using Model Finder (Kalyaanamoorthy et al. 2017) based on the Bayesian information criterion. Genetic distances among the sequences were calculated using the Kimura 2-parameter (K2P) distance model in MEGA 7. The resulting ML tree was visualized using FIGTREE v. 1.4.3. Bayesian-inference (BI) analysis was performed in MRBAYES v. 3.2.7 using two independent Markov Chain Monte Carlo chains for 50,000,000 generations, with the first 25% of trees discarded as burn-in. The remaining trees were combined to produce a 50% majority-rule consensus tree. Posterior probabilities for each clade were calculated from BI, with values exceeding 0.95 considered significant support. Estimates of evolutionary divergence among sillaginid species were derived using the Kimura 2-parameter (K2P) model implemented in MEGA 11 (Tamura et al. 2021).

To delineate species, we applied three molecular species delimitation methods: (a) two distance-based methods, Automatic Barcode Gap Discovery (ABGD) and Assemble Species by Automatic Partitioning (ASAP); and (b) a topology-based method, Bayesian Poisson Tree Process (bPTP). The COI sequence dataset was analyzed on the ABGD webserver https://bioinfo.mnhn.fr/abi/public/abgd/abgdweb.html with a combination of settings in the parameter range of Pmin = 0.001, Pmax = 0.1, and gap width = 0.1–0.9, across a total of 10 steps, applying a K2P-corrected genetic distance matrix. For ASAP, the partition score integrates two metrics: barcode gap width and the probability of panmixia (*p*-value), where a lower score indicates a more optimal partition. This method was executed using website https://bioinfo.mnhn.fr/abi/public/asap/asapweb.html with the Kimura (K80) model.

## ﻿Results

### ﻿Taxonomic account

**Family Sillaginidae Richardson, 1846**

**Genus *Sillago* Cuvier, 1817**

#### ﻿ Sillago persica

Taxon classificationAnimaliaPerciformesSillaginidae

34BBE2EF-79D7-5910-911C-58EB6EEF62DC

https://zoobank.org/18029739-2FD6-4E76-8669-3DC17FBF6C05

Fig. 2, Table 1

##### Type material.

***Holotype***: • TAC1245F; 165 mm SL; sex unknown sex; Iran, Bushehr Province; 28°54'N, 50°46'E; Hashem Khandan Barani leg.; December 2021.

***Paratypes***: • TAC1246F; 30 specimens, 114–195 mm TL, weight 11.6–35.9 g; Iran, Bushehr Province; located in the northern Persian Gulf, Iran; Hashem Khandan Barani leg.; December 2021.

##### Etymology.

The species name *persica* is derived from the Persian Gulf, where the type specimens were collected.

##### Diagnosis.

Dorsal fin with XI, I+21–22 rays and anal with II (23–24) rays. The lateral line with 65–76 scales, 4–5 scale rows between the dorsal-fin origin and the lateral line. Gill rakers with 3+7–8 on the first gill arch. The vertebral formula with 14 or 15 abdominal vertebrae (predominantly 14), 4–5 predorsal vertebrae (mostly 4), and 14–15 caudal vertebrae (mostly 14), resulting in a total vertebral count of 32–34 (mostly 32) (Table 1). The body is characterized by the absence of dark blotches or a mid-lateral stripe. The swim bladder features two posterior extensions, with a duct-like process originating from the anterior end of the swim bladder and beginning at the junction of the roots of the two posterior extensions (Fig. 3). Five wide extensions connect the posterior sub-extensions of the anterolateral extensions to the body of the swim bladder.

**Table 1.** Morphometric measurements and counts of *Sillago persica*.

Meristic and Morphometric Measurements (mm)	Sillago persica	
	Holotype	Paratypes (n = 40)
Total weight (g)	30.81	11.63–31.94
Total length	165	114–175
Standard length	145	101–154
Head length	41	29–43
Snout length	18	11–19
Eye diameter	7	6–8
Interorbital width	8	5–8
Postorbital length	19	11–20
Body depth	23	17–24
Body width	18	14–20
Length of caudal peduncle	8.5	8–9
Depth of caudal peduncle	9	6–10
Base of the 1^st^ dorsal-fin	28	21–30
Base of the 2^nd^ dorsal-fin	47	37–49
Base of the anal-fin	50	39–54
Pectoral-fin length	22	15–23
Pelvic-fin length	18	14–19
Vertebrae	32	32–34
Dorsal-fin rays	XI, I + 21	XI, I + (21–22)
Pectoral-fin rays	16	15–16
Pelvic-fin rays	I + 5	I + (5–6)
Anal-fin rays	II + 23	II + (23–24)
Caudal-fin rays	16	16 (16–18)
Gill rakers on first arch	3 + 8	(3) + (7–8)
Lateral line scales	71	65–76
Scales above/below lateral line	4/9	(4–5)/(9–10)
**As % of SL**		
Body depth	15.8	14.92–16.83
Head length	28.7	26.35–28.71
**As % of HL**		
Eye diameter	17.07	15.38–21.95
Interorbital width	19.5	17.24–20.59
Snout length	43.9	37.93–44.19
Postorbital length (PL)	46.3	37.93–50.12

##### Description.

**Figure 2.**
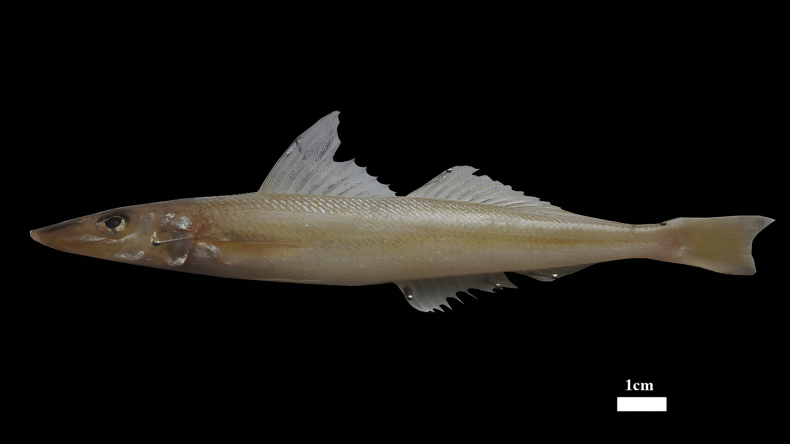
*Sillago persica*, sp. nov., holotype (TAC1245F), 165 mm SL, Persian Gulf coast, Iran.

General body features are illustrated in Fig. 2, and counts and measurements are provided in Table 1. The body is elongate, slightly conical at the anterior end, and tubular towards the posterior end. The body depth is 14.9 mm, 16.83% of SL. The head is large, and its length is 26.3–28.7% of SL. The snout is long, and its length is 38.2–45.8% of HL. The eye is of moderate size, and its diameter is 15.3–17.6% of HL. The interorbital region is flat, and its width is 17.9–21.1% of HL. The mouth is small, terminal, and the tips of the upper and lower jaws are almost equal in length. Both jaws have a series of very small teeth that which form a broad band that tapers posteriorly into a single row. Gill rakers on the first arch are pointed and relatively large. The caudal peduncle is short; its depth is 77.5–90.1% of the caudal-peduncle length. The body is covered in small to moderate-sized ctenoid scales, while the cheeks are covered in cycloid scales. The lateral line begins above the gill aperture and the anterior portion of the pectoral fin, extending along the dorsal edge to the end of the body.

**Figure 3.**
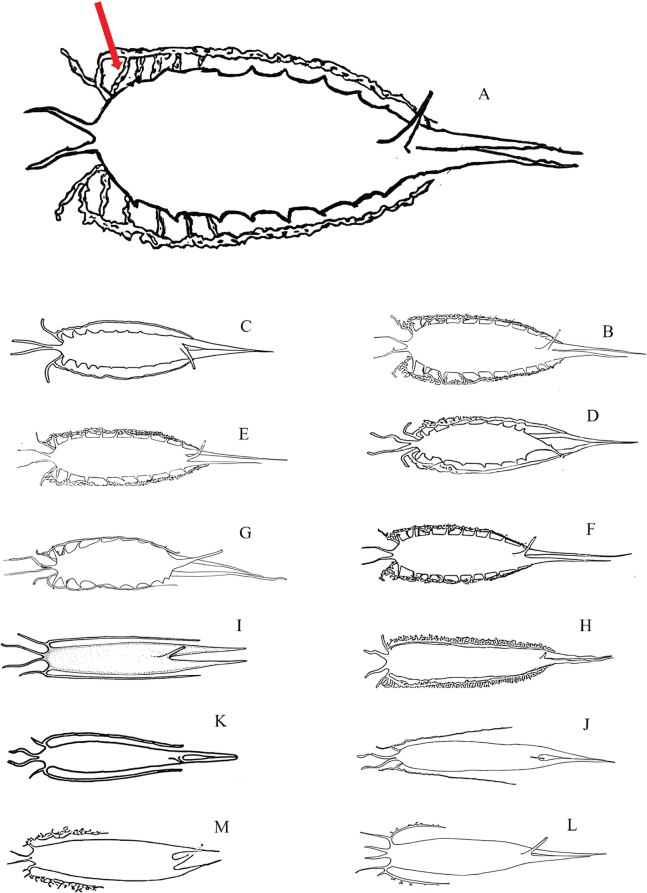
Swim bladders of 13 *Sillago* species with two posterior extensions. Red arrow indicated to five extensions connect the posterior sub-extensions of the anterolateral extensions to the body of swim bladder. A. *S. persica* sp. nov.; B. *S. sihama*; C. *S. mengjialensis*; D. *S. muktijoddhai*; E. *S. shaoi*; F. *S. nigrofasciata*; G. *S. indica*; H. *S. parvisquamis*; I. *S. intermedius*; J. *S. caudicula*; K. *S. suezensis*; L. *S. sinica*; M. *S. parasihama* (Sources: Cheng et al. 2021; Saha et al. 2024).

The species has two separate dorsal fins, with rays as XI, I+21 (range: 21–22), and the fin membrane has blurry black spots. The anal fin has II, 23 (range: 23–24) rays. The pectoral fin with 15 (15–16) rays. The pelvic fins are separate, wide, and have I+5 rays, approximately triangular and smaller than the pectoral fins. The caudal fin has 16–18 rays.

Color of fresh specimens: the upper surface of the head is bright brown, while the trunk is also bright brown, transitioning to silver on the abdomen. The body lacks stripes, although the anterior half of the ventral side may exhibit some dark pigmentation. The dorsal fins are hyaline, featuring small dark spots on the fin membrane. The pectoral and pelvic fins are light yellowish hyaline; the anal fin is hyaline and free of dark spots, and the caudal fin is yellowish dusky, with a black margin along the posterior edge.

Swim bladder: the swim bladder is large and features two anterior extensions that extend forward to the basioccipital, which is positioned above the auditory capsules on both sides. Additionally, it has two posterior tapering extensions that reach into the caudal region without a lacuna between them. Two anterolateral extensions arise from the anterior portion of the swim bladder, each bifurcating into anterior and posterior sub-extensions. The anterior sub-extension consists of a short, simple, blind tubule, while the posterior sub-extensions are kinked, long, and complex at the beginning, becoming simpler and relatively thinner as they extend along the abdominal wall, ultimately terminating at the base of the posterior extensions. Notably, five wide extensions connect to these two sub-extensions, a feature that is not observed in other species within this genus. Furthermore, a single duct-like process originates from the pelvic surface of the swim bladder, extending to the urogenital opening. This duct-like process originates at the junction of the roots of the two posterior extensions, located anterior to the terminus of the swim bladder.

##### Distribution.

In this study, specimens of *S. persica* were collected from the northern Persian Gulf coast of Iran (Bushehr) (Fig. 1). The identified haplotypes formed a monophyletic group alongside the haplotypes of the G5 clade of *S. sihama*, which were collected from the coast of Karachi, Pakistan (Cheng et al. 2021). Thus, the known distribution of this species extends from the northern coast of the Persian Gulf to the northern coast of the Arabian Sea.

##### Comparison.

According to the subgenera grading system for the genus *Sillago* proposed by McKay (1985), *S. persica* is classified within the subgenus Sillago (Sillago) due to the presence of two posterior extensions of the swim bladder. This study validates the recognition of *S. persica* as a new species through comparisons with all species in this genus. Distinctions among these species can primarily be made based on the meristic and morphometric characters outlined in Table 2. The new species is immediately distinguished from others by below several key characters: it has a smaller head than *S. megacephalus* (26.35–28.71% SL vs 33%). It has more branched anal fin rays than *S. parasihama*, *S. shaoi*, *S. muktijoddhai*, *S. nigrofasciata*, and *S. suezensis* (23–24 vs 19–21). It has fewer scale rows between the lateral line and dorsal-fin origin than *S. intermedius* (4–5 vs 6–7) and a smaller head (26.3–28.71% SL vs 30.0–31.0%). It has fewer lateral line scales than *S. parvisquamis* (65–76 vs 79–84). It has fewer transversal scale rows than *S. parvisquamis* (4–5/8–10 vs 7/11–12). It has fewer a total of vertebrae than *S. shaoi*, *S. parvisquamis*, *S. caudicula*, and *S. sinica* (32–34 vs 35–40).

**Table 2.** Comparison of *Sillago persica* sp. nov. and 15 other species of *Sillago* with two posterior extensions of the swim bladder. XI, I, 20–22.

Species	Dorsal-fin rays	Anal-fin rays	Pectoral-fin rays	Pelvic-fin rays	Scales in lateral line	Scales above/ below lateral line	Gill rakers first arch	Vertebrae	HL/SL (%)
*S. persica* ^a^	XI, I, 21–22	II, 23–24	15–16	I + (5–6)	65–76	4–5/9–10	3/7–8	32–34	26.35–28.71
*S. malabarica* ^b^	XI–XII, I, 21–24	II, 22–24			68–72	4–5/8–9.5	3–4/6–8	34	25–30.4
*S. intermedius* ^c^	XI, I, 21–22	II, 21–22			67–70	6–7/8–9	-	34	30.0–31.0
*S. megacephalus* ^c^	XI, I, 22	II, 23			70	5/10–11	-	-	33.0
*S. panhwari* ^d^	X–XII, I, 20–22	II, 18–23			69–84	4–5/7–10	3–4/7–8	34	27.9–35.0
*S. shaoi* ^e^	XI, I, 20–22	II, 21–22	16–18	I + 5	70–73	5–6/10–12	3–4/5–6	35	26.1–31.0
*S. parvisquamis* ^c^	XII–XIII, I, 20–22	II, 22–24			79–84	7/11–12	1–2/7–9	39–40	25.9–27.7
*S. sihama* ^e^	XI, I, 20–23	II, 21–23			68–72	5–6/10–12	3/8–9	34	24.0–30.0
*S. caudicula* ^f^	XI, I, 22–23	II, 23–24			71	5/11	4/11	35–36	29.0–30.1
*S. sinica* ^g^	X–XI, I, 20–22	II, 21–23			75–79	5–6/9–11	2–4/6–8	37–39	24.7–29.8
*S. suezensis* ^h^	X–XII, I, 19–22	II, 18–22			63–74	-	3–4/8–10	34	26.6–27.0
*S. indica* ^i^	X–XI, I, 20–22	II, 21–23			68–71	5–6/10–12	3–4/7–8	33–35	27.5–32.4
*S. muktijoddhai* ^j^	X–XI, I, 20–22	II, 21–23	15–18	I + 5	68–72	4–6/10–13	3–5/8–10	32–35	21.8–31.3
*S. mengjialensis* ^j^	XI, I, 20–22	II, 20–23	15–17	I + 5	66–72	4–5/10–12	3–4/8–10	31–35	25–31.7
*S. nigrofasciata* ^k^	X–XII, I, 20–22	II, 20–22	14–16	I + 5	67–75	4–6/9–12	2–4/5–8	34–35	25.1–30.8
*S. parasihama* ^l^	XI–XII, I, 18–21	II, 19–21	16–17	I + 5	65–70	4–5/8–10	2–3/5–7	34	18.4–29.0

Notes: a, Тhis study; b, Divya et al. 2021; c, McKay (1985, 1992); d, Panhwar et al. (2018); e, Xiao et al. (2016); f, Kaga et al. (2010); g, Gao et al. (2011); h, Golani et al. (2014); i, Kaga and Ho (2012); j, Saha et al. (2022); k, Xiao et al. (2021); l, Yu et al. (2022).

A comparison of swim bladder characteristics, a key trait for species diagnosis within this family, revealed that the swim bladder of *S. persica* is very similar to that of *S. sihama*, *S. nigrofasciata*, *S. shaoi*, *S. muktijoddhai*, and *S. mengjialensis*. However, notable differences exist. In *S. shaoi* and *S. muktijoddhai*, the roots of the two posterior extensions are non-adjacent, with a lacuna present between them, whereas in *S. persica*, *S. nigrofasciata*, *S. mengjialensis*, and *S. sihama*, the roots of the two posterior extensions are adjacent and lack a lacuna. Furthermore, the posterior sub-extension of the anterolateral extensions in *S. persica* is distinctive, characterized by a complex structure at its beginning, where five wide extensions connect to these two sub-extensions, a feature not observed in *S. nigrofasciata*, *S. mengjialensis*, and *S. sihama.* Additionally, the origin of the duct-like process in *S. persica* differs from that in *S. parasihama* and *S. indica*. In *S. persica*, the duct-like process originates anterior to the terminus of the swim bladder and before the junction of the roots of the two posterior extensions. In contrast, the duct-like process in *S. parasihama* and *S. indica* originates at the terminus of the swim bladder and arises at the junction of the roots of the two posterior extensions.

Furthermore, among the 12 known species of *Sillago* with two posterior extensions, *S. persica* can be easily distinguished from *S. caudicula* and *S. intermedius* based on body coloration, as both *S. intermedius* and *S. caudicula* exhibit dusky black spots on their bodies. It can also be differentiated from *S. parvisquamis* and *S. sinica* by the presence of dusky spots on the second dorsal-fin membranes, which show five or six rows in *S. parvisquamis* and three or four rows in *S. sinica.* Additionally, *S. persica* can be empirically distinguished from *S. nigrofasciata*, *S. indica*, and *S. shaoi* by the coloration of the anal fin: *S. persica* has a hyaline anal fin without black spots, whereas *S. nigrofasciata* typically has a yellowish anal fin with sparse black spots, and *S. indica* and *S. shaoi* have yellowish-brown anal fins with black dots on the interradial membranes. Moreover, *S. indica*, *S. suezensis*, and *S. panhwari* possess a faint midlateral stripe on their bodies, which is not present in *S. persica*.

##### Genetic analysis of the COI gene.

**Figure 4.**
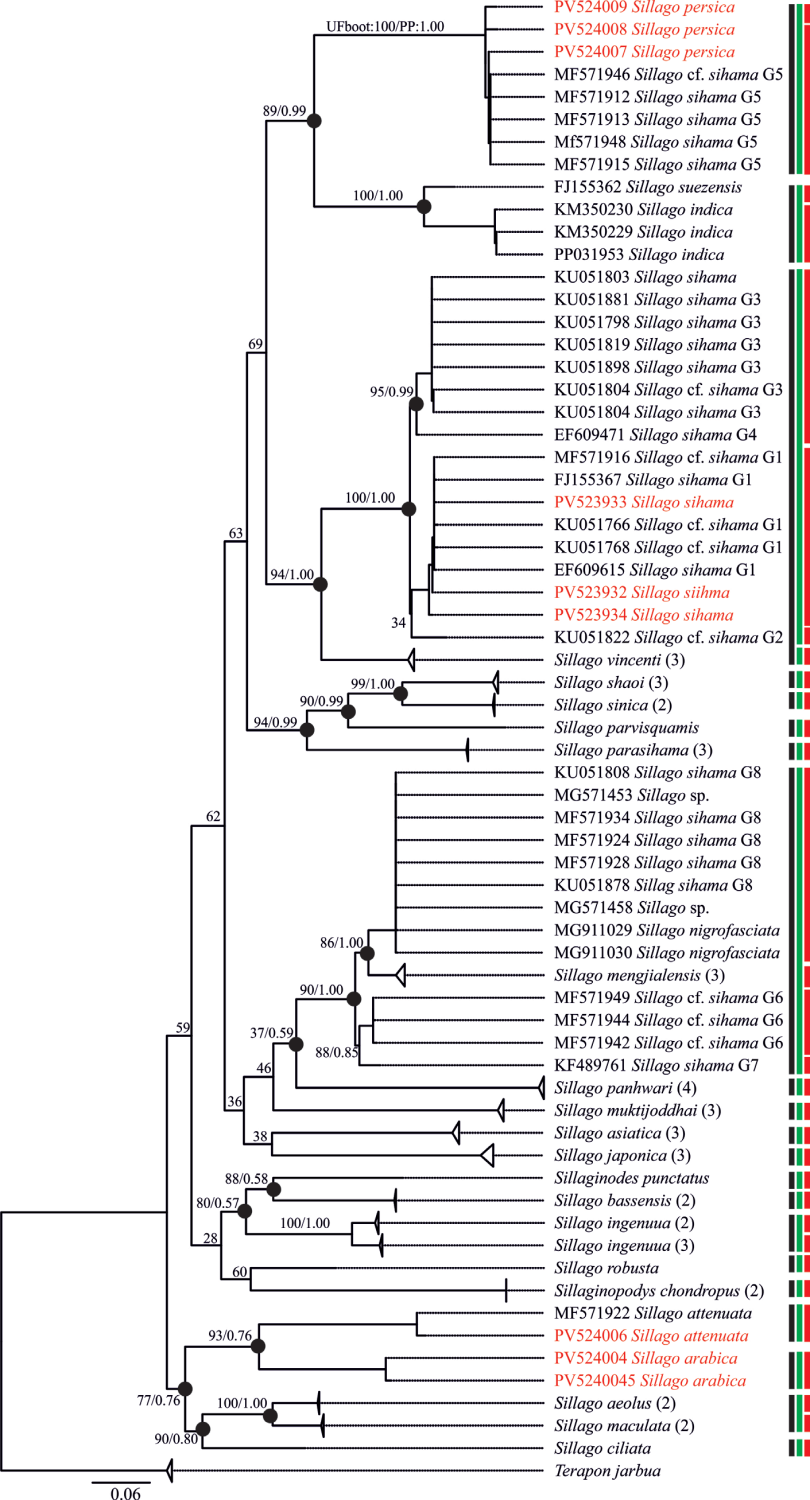
Phylogeny recovered using the maximum-likelihood (ML) and Bayesian-inference (BI) analyses of the COI dataset. The black circles indicate congruent nodes with ML and BI analyses. UltraFast Bootstrap support and posterior probabilities are written above congruent branches. Three species delimitation methods are represented by black (ASAP), green (ABGD), and red (bPTP). The numbers in parentheses indicate the number of sequences that were collapsed.

Sixrty-three sequences from *Sillago* species were utilized in the genetic analysis. The genetic distances among sillaginid species ranged from 0.05 to 0.26 K2P, while the genetic divergences within known species were between 0.00 and 0.01 K2P. The genetic distance between *S. persica* and other recognized *Sillago* species varied from 0.18 to 0.024 K2P. In comparison with the previous eight clades of the *S. sihama* complex, the genetic distance to clade G5 was 0.01, while distances to the other eight clades were 0.18–0.20 (Table 3; Suppl. material 1). The Bayesian and maximum-likelihood phylogenetic tree based on COI gene sequences (Fig. 4) indicated that the three haplotypes of *S. persica* and five haplotypes from the *S. sihama* clade G5 (MF571946; Cheng et al. 2021) formed a monophyletic group. This clade is positioned as a sister group to a clade consisting of three haplotypes of *S. indica* and one haplotype of *S. suezensis.* Additionally, the three haplotypes from specimens identified as *S. sihama* in this study (PV523932–PV523934) clustered in a monophyletic group with those from *S. sihama* clade G1 (Cheng et al. 2021). The results from three species delimitation models (ASAP, ABGD, and bPTP) further support the classification of *S. persica* haplotypes as a distinct species.

**Table 3.** Genetic distances (K2P) among and within haplotypes of five sillanigid species in the Persian Gulf and the Oman Sea area, haplotypes of eight clades of *Sillago sihma* (G1–G8 reported by Cheng et al. 2021), and the haplotypes of *Sillago persica* in the present study (PV524007–PV524009).

Species	1	2	3	4	5	6	7	8	9	10	11	12	13	14
**1** *S. persica*	**0.01**													
**2** *S. arabica*	0.19	**0.00**												
**3** *S. attenuata*	0.23	0.19	**0.01**											
**4** *S. indica*	0.21	0.21	0.23	**0.00**										
**5** *S. panhwari*	0.24	0.22	0.22	0.21	**0.00**									
**6***S. sihama*-G1	0.20	0.21	0.22	0.18	0.20	**0.00**								
**7***S. sihama*-G2	0.19	0.19	0.21	0.18	0.21	0.05	**n/c**							
**8***S. sihama*-G3	0.19	0.21	0.22	0.17	0.21	0.04	0.04	**0.00**						
**9***S. sihama*-G4	0.19	0.20	0.21	0.16	0.20	0.03	0.04	0.02	**n/c**					
**10***S. sihama*-G5	0.01	0.19	0.23	0.19	0.23	0.20	0.19	0.19	0.19	**0.00**				
**11***S. sihama*-G6	0.19	0.18	0.21	0.21	0.18	0.18	0.17	0.18	0.18	0.19	**0.00**			
**12***S. sihama*-G7	0.18	0.17	019	0.22	0.16	0.18	0.17	0.19	0.19	0.19	0.03	**n/c**		
**13***S. sihama*-G8	0.20	0.19	0.21	0.22	0.20	0.18	0.17	0.18	0.17	0.20	0.06	0.06	**0.00**	
**14** *Sillaginopodys chondropus*	0.24	0.20	0.20	0.23	0.23	0.20	0.21	0.21	0.20	0.24	0.23	0.21	0.22	**0.00**

## ﻿Discussion

This study introduces a new species, *Sillago persica*, which is characterized through both morphological and DNA-barcoding methods. Genetic analysis based on COI sequences from the sillaginid species indicated that the genetic divergences between *S. persica* and other sillaginid species as well as clades of *S. sihama* ranged from 0.18 to 0.24 K2P. These values are significantly higher than the intraspecific genetic distances, which ranged from 0.00 to 0.01 K2P.

*Sillago persica* shares similarities with *S. sihama* in terms of countable characters and swim bladder structure; however, it is distinctly different from *S. sihama* due to the shape of its swim bladder and a significant genetic divergence of 0.18–0.20 K2P (Compared with different clades of *S. sihama* complex except G5, Table 3). The posterior sub-extensions of *S. persica* are characterized by unique transverse appendages that are attached to the body of the swim bladder, a feature not found in other species. Furthermore, based on the morphometric and meristic characters, *S. persica* can be differentiated from the other 15 *Sillago* species (refer to Table 2). Notably, the number of soft rays in the anal fin serves as a distinguishing character that separates this species from *S. parasihama*, *S. shaoi*, *S. muktijoddhai*, *S. mengjialensis*, *S. nigrofasciata*, and *S. suezensis.*

Currently, there are 39 recognized valid species within the family Sillaginidae (Saha et al. 2024). Five of these species have been documented in the northern coast of the Persian Gulf and the Oman Sea, Iran: *S. sihama*, *S. arabica*, *S. attenuata*, *S. indica*, and *Sillaginopodys chondropus* (McKay 1992; Alavi-Yeganeh et al. 2016; Khandan Barani et al. 2023;). The taxonomic placement of the newly described species can be assessed based on the morphology of the swim bladder, which serves as a key diagnostic feature within the family. Variations in swim bladder structure are commonly used to define subfamilies and genera. Comparing the new species’ swim bladder, especially the five extensions linking posterior sub-extensions to the main body, with those of known taxa allows inference of its phylogenetic position and subfamily. This approach provides a more robust understanding of the species’ evolutionary relationships within the Sillaginidae and contributes to resolving taxonomic ambiguities in the region.

Within the genus *Sillago*, *S. sihama* displays significant genetic diversity across the Indo-West Pacific region. Several recently described *Sillago* species in this area include *S. caudicula* (from Oman and Madagascar), *S. sinica* (from China), *S. suezensis* (from the northern Red Sea and Mediterranean), *S. shaoi* (from the Taiwan Strait), *S. panhwari* (from the northern Arabian Sea), *S. nigrofasciata* (from the southern coast of China), *S. parasihama* (from Beihai and Zhanjiang, China), *S. muktijoddhai*, and *S. mengjialensis* (from the Bay of Bengal, Bangladesh). These species have often been misidentified as *S. sihama* (Kaga et al. 2010; Gao et al. 2011; Kaga and Heemstra 2013; Golani et al. 2014; Panhwar et al. 2018; Xiao et al. 2021; Saha et al. 2022; Yu et al. 2022). *Sillago persica* is a new species that has been distinguished from *S. sihama.* The description of this new *Sillago* species from the northern Persian Gulf, along the coast of Iran, highlights the need for further investigation into its ecology, distribution, and abundance patterns. Such information is essential for the effective implementation of management practices and the conservation of ecologically and economically important sillaginids in this region.

## Supplementary Material

XML Treatment for Sillago persica

## ﻿Additional information

### Use of AI

No use of AI was reported.

### Author ORCIDs

Hashem Khandan Barani https://orcid.org/0000-0003-4913-5750

Mohammad Sadegh Alavi-Yeganeh https://orcid.org/0000-0002-6246-9504

Mehdi Ghanbarifardi https://orcid.org/0000-0002-1825-2551

### Data availability

All of the data that support the findings of this study are available in the main text or Supplementary Information.

## Supplementary materials

Supplementary material 1 Genetic distances (K2P) Data type xls Explanation note Genetic distances (K2P) among and within haplotypes of 24 sillanigid species, eight clades of *Sillago sihama* complex and the haplotypes of *Sillago persica*. https://binary.pensoft.net/file/1434649This dataset is made available under the Open Database License (http://opendatacommons.org/licenses/odbl/1.0/). The Open Database License (ODbL) is a license agreement intended to allow users to freely share, modify, and use this Dataset while maintaining this same freedom for others, provided that the original source and author(s) are credited.Hashem Khandan Barani, Mohammad Sadegh Alavi-Yeganeh, Mehdi Ghanbarifardi
